# Isolation and Potential for Transmission of *Mycobacterium bovis* at Human–livestock–wildlife Interface of the Serengeti Ecosystem, Northern Tanzania

**DOI:** 10.1111/tbed.12445

**Published:** 2015-11-13

**Authors:** B. Z. Katale, E. V. Mbugi, K. K. Siame, J. D. Keyyu, S. Kendall, R. R. Kazwala, H. M. Dockrell, R. D. Fyumagwa, A. L. Michel, M. Rweyemamu, E. M. Streicher, R. M. Warren, P. van Helden, M. I. Matee

**Affiliations:** ^1^ Department of Microbiology and ImmunologySchool of MedicineMuhimbili University of Health and Allied Sciences (MUHAS)Dar es SalaamTanzania; ^2^ Tanzania Wildlife Research Institute (TAWIRI)ArushaTanzania; ^3^ DST/NRF Centre of Excellence for Biomedical Tuberculosis Research/SAMRC Centre for Tuberculosis ResearchDivision of Molecular Biology and Human GeneticsFaculty of Medicine and Health SciencesStellenbosch UniversityTygerbergCape TownSouth Africa; ^4^ Centre for Emerging, Endemic and Exotic diseasesRoyal Veterinary College (RVC)Hawkshead LaneNorth MymmsHatfieldHertfordshireUK; ^5^ Department of Veterinary Medicine and Public HealthFaculty of Veterinary MedicineSokoine University of Agriculture (SUA)MorogoroTanzania; ^6^ Department of Immunology and InfectionLondon School of Hygiene and Tropical Medicine (LSHTM)LondonUK; ^7^ Department Veterinary Tropical DiseasesFaculty of Veterinary ScienceUniversity of PretoriaOnderstepoortSouth Africa; ^8^ Southern African Centre for Infectious Diseases Surveillance (SACIDS)Sokoine University of Agriculture (SUA)Chuo KikuuMorogoroTanzania

**Keywords:** *Mycobacterium bovis*, spoligotype, MIRU‐VNTR, human–animal interface, serengeti ecosystem

## Abstract

*Mycobacterium bovis*, the causative agent of bovine tuberculosis (bTB), is a multihost pathogen of public health and veterinary importance. We characterized the *M. bovis* isolated at the human–livestock–wildlife interface of the Serengeti ecosystem to determine the epidemiology and risk of cross‐species transmission between interacting hosts species. DNA was extracted from mycobacterial cultures obtained from sputum samples of 472 tuberculosis (TB) suspected patients and tissue samples from 606 livestock and wild animal species. *M. bovis* isolates were characterized using spoligotyping and Mycobacterial Interspersed Repetitive Units‐Variable Tandem Repeats (MIRU‐VNTR) on 24 loci. Only 5 *M. bovis* were isolated from the cultured samples. Spoligotyping results revealed that three *M. bovis* isolates from two buffaloes (*Syncerus caffer*) and 1 African civet (*Civettictis civetta*) belonged to SB0133 spoligotype. The two novel strains (AR1 and AR2) assigned as spoligotype SB2290 and SB2289, respectively, were identified from indigenous cattle (*Bos indicus*). No *M. bovis* was detected from patients with clinical signs consistent with TB. Of the 606 animal tissue specimens and sputa of 472 TB‐suspected patients 43 (7.09%) and 12 (2.9%), respectively, yielded non‐tuberculous mycobacteria (NTM), of which 20 isolates were *M. intracellulare*. No *M. avium* was identified. *M. bovis* isolates from wildlife had 45.2% and 96.8% spoligotype pattern agreement with AR1 and AR2 strains, respectively. This finding indicates that bTB infections in wild animals and cattle were epidemiologically related. Of the 24 MIRU‐VNTR loci, QUB 11b showed the highest discrimination among the *M. bovis* strains. The novel strains obtained in this study have not been previously reported in the area, but no clear evidence for recent cross‐species transmission of *M. bovis* was found between human, livestock and wild animals.

## Introduction

Bovine tuberculosis (bTB) is a bacterial zoonosis caused by *Mycobacterium bovis*. The emergence of zoonotic diseases in human‐livestock‐wildlife interface areas has been primarily facilitated by anthropogenic changes due to agriculture and human pressure (Rhyan and Spraker, 2010). Cattle are the principal domestic animal host for *M. bovis,* and buffalo are recognized as wildlife maintenance hosts in most African countries (Cousins, 2001). The presence of wildlife reservoirs acting as spillover hosts for *M. bovis* in human–animal interface areas has interfered with the eradication of bTB in many countries (Thoen et al., 2009).

In Tanzania, infection due to *M. bovis* has been reported in a range of hosts including cattle (Kazwala et al., 1998; Shirima et al., 2003; Mdegela et al., 2004; Cleaveland et al., 2007; Durnez et al., 2009; Mwakapuja et al., 2013a), humans (Mfinanga et al., 2004) and wildlife species (Cleaveland et al., 2005). Evidence for interspecies transmission is supported by the genetic relatedness between *M. bovis* isolated from infected wildlife and local livestock or between livestock and man (Kazwala et al., 2006; Clifford et al., 2013). A representative set of samples of *M. bovis* isolated from cattle and man showed a high degree of genetic diversity which was distributed throughout Tanzania, possibly reflecting extensive internal movements of cattle belonging to pastoralists (Kazwala et al., 2006). It has been proposed that livestock movements may increase opportunities for cross‐species transmission between wild and domestic species (Cunha et al., 2012). The epidemiology of bTB in wildlife is said to be partly driven by resource selection and spatial ecology of wild herbivores sharing common resources (de Garine‐Wichatitsky et al., 2013). Spillover of bTB from livestock to wildlife or humans, and spillback from wildlife to livestock are influenced by environmental conditions, human behaviour and type of interface (de Garine‐Wichatitsky et al., 2013).

The Serengeti is one of the largest conservation areas in the world and is particularly important as it is one of a very few areas where natural seasonal herbivore migration occurs. It is a major tourist attraction and an important area for research into savannah ecosystems. There is no fencing in the Serengeti ecosystem and therefore livestock from neighbouring pastoralists and agropastoralists interact with wild animals at water sources or when they move close to wildlife conservation areas searching for pasture (Katale et al., 2012), mostly during the dry season. The interactions at this human–livestock–wildlife interface area of the ecosystem increase the chances of interspecies transmission of pathogens. Human pressure due to encroachment and farming activities close to protected areas in addition to migrating ungulates might further facilitate cross‐species disease transmission. The main risk factors leading to introduction and maintenance of infection in a wild animal population have been identified in South Africa (Renwick et al., 2007) and the rest of the world (Rhyan and Spraker, 2010; de Garine‐Wichatitsky et al., 2013). They include species diversity (maintenance or spillover hosts), social behaviour of wildlife hosts, wildlife densities (threshold population/community densities), movements of animal populations (possible introduction through migratory individuals or confinement), and the absence or inefficiency of wildlife bTB surveillance and control (de Garine‐Wichatitsky et al., 2013). All these risk factors may characterize the Serengeti ecosystem. However, it can be argued that the lack of bTB surveillance and control does not only apply to wildlife species, but also to livestock. The Serengeti ecosystem is characterized by high indirect contacts between livestock and wildlife (Katale et al., 2013), which favours disease transmission between species. However, strain diversity of *M. bovis* and its potential dynamics of dissemination across species in the ecosystem have not been fully explored. Moreover, the husbandry practices, proximity to wildlife and tradition customs of consuming raw meat/milk by the pastoralist community in the ecosystem, epidemiological studies are needed to explore the disease dynamics in the ecosystem and bTB infection in animals and ultimately also humans (Katale et al., 2013).

Spoligotyping, a genotyping method based on the characterization of the DR locus present in all the members of the *M. tuberculosis* complex has been useful in differentiation of mycobacteria and provides valuable epidemiological information on strain identities (Gori et al., 2005). The introduction of analysis of mycobacterial interspersed repetitive units (MIRU) based on variable‐number tandem repeats (VNTRs) of genetic elements has become an important method for epidemiological studies, as it allows high‐throughput, discriminatory and reproducible analysis of clinical isolates (Supply et al., 2006). When MIRU‐VNTR analysis of isolates using 24 loci are combined with spoligotyping, this combination may exceed the discrimination offered by restriction fragment length polymorphism (RFLP) (Christianson et al., 2009). Furthermore, MIRU‐VNTR can help understand epidemiological links and therefore evaluate pattern of disease whether epidemic or resulting from re‐infection thus defining a transmission chain (Hilty, 2006; Supply et al., 2006). Using spoligotyping and MIRU‐VNTR analysis, this study aimed to investigate *M. bovis* diversity and whether bTB transmission is occurring across species at the human–animal interface of the Serengeti ecosystem.

### Study site

**Figure 1:**
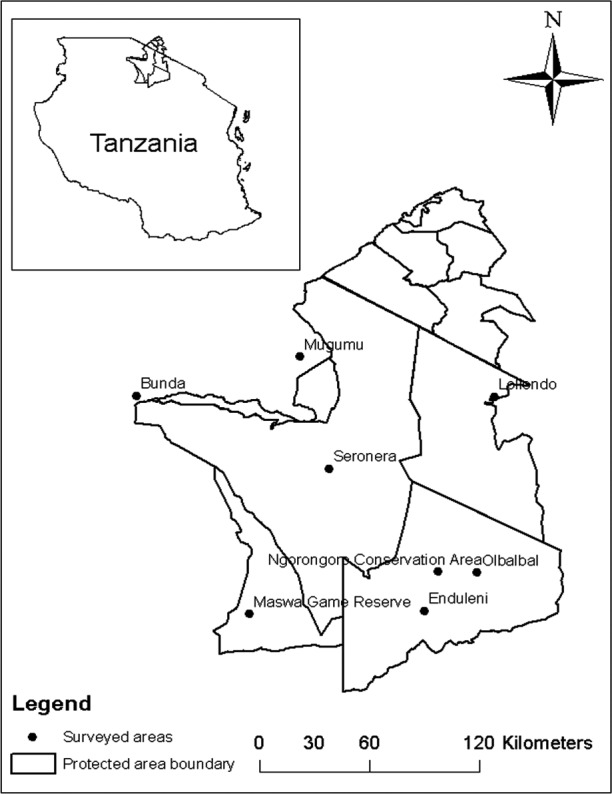
Map of the Serengeti ecosystem showing the distribution of M. bovis spoligotype and study sites where animal tissues and human sputum samples collected at slaughter houses and hospitals respectively.

This study was conducted in the Serengeti ecosystem comprising the Serengeti National Park (SNP), Ngorongoro Conservation Area (NCA), Loliondo Game Controlled Area (LGCA), Maswa Game Reserve and Ikorongo‐Grumeti Game Reserve (IGGR), and surrounding districts of Bunda (2°0′ 0″S; 33°49′60″E), Serengeti (2°0′0″S; 34°49′60″E) and Ngorongoro (2°S and 4°S; 35°E and 36°E) (Fig. 1). The ecosystem spans some 30 000 km^2^ in northern Tanzania and extends to south‐western Kenya between latitudes 1° and 4°S and longitudes 34° and 36°E. The NCA to the east is a unique multiple land‐use area where human, livestock and wildlife legally coexist. The ecosystem is featured by annual movements of ungulates, mostly blue wildebeest (*Connochaetes taurinus*) and zebra (*Equus burchelli*) interacting with other fauna while crossing over the ecosystem from northern Tanzania to the Maasai Mara National Reserve in south‐western Kenya. The ecosystem is unique in the sense that human, livestock and wildlife coexist (Ngorongoro conservation area, part of the Serengeti ecosystem). Moreover, traditional customs of pastoralists including the Maasai who consume raw milk/meat predispose them to zoonotic diseases. Among which, might be tuberculosis. The north‐western side of the Serengeti ecosystem is dominated by agropastoral communities which depend on subsistent agriculture and livestock as source of income (Katale et al., 2013). The eastern side is dominated by Maasai pastoralists who depend to a large extent on livestock as a source of income. Livestock and wild animals in the ecosystem are reported to interact while grazing and watering which may increase the possibility of interspecies disease transmission (Katale et al., 2013).

### Study design

#### Sampling of livestock and wildlife tissues

##### Livestock

Livestock sampling was carried out in the slaughter houses located in district headquarters. Cattle were purchased from different local markets and transported to the slaughter houses which were also close to wildlife sampling areas. Meat inspections were conducted with assistance from meat inspectors. Tissues samples presenting TB‐like lesions from four hundred and ninety nine (499) indigenous cattle (*Bos indicus*) were obtained from different sites, including mediastinal, retropharyngeal, pre‐crural and pre‐scapular lymph nodes, lungs and liver. Tissues were collected from approximately 1200 slaughtered indigenous zebu in Serengeti (420 tissues), Bunda (600 tissues) and Ngorongoro (180 tissues) abattoirs. Of the 499 tissues with suggestive of mycobacterial infection 205, 220 and 74 tissues were collected from Serengeti, Bunda and Ngorongoro districts, respectively. To ensure that only cattle carcasses from the study site were sampled, the history of the origin of the cattle was taken from the owners. The anatomical sites of bTB‐suspected lesions for each carcass were recorded. With assistance from meat inspectors, tuberculous organs and their associated lymph nodes were palpated, incised and preserved separately in sterile zip lock bags and packed in a cool box before processing for storage in liquid nitrogen (LN). Tissues stored in LN were safely transported to the laboratory at Sokoine University of Agriculture (SUA), Tanzania, for initial processing and culture.

##### Wildlife

Tissues from 107 wild animals were sampled in the Serengeti ecosystem (Table 1) in the period from 2010 to 2013. Tissues such as mediastinal lymph nodes, retropharyngeal lymph nodes, lungs, mesenteric lymph nodes, liver and kidney were collected aseptically during trophy and meat cropping in Maswa Game Reserve (MGR) coordinated by the Ministry of Natural Resources and Tourism (MNRT), Tanzania. Mediastinal and retropharyngeal lymph nodes were opportunistically collected from dead animals and stored in LN containers for subsequent analysis at SUA. Only tissues from intact internal organs were sampled from road kill animals.

**Table 1:** Culture and Mycogenus PCR results of 606 wild animals and cattle tissues collected in the Serengeti ecosystem

Species of animal tested	Total no of animal tissues tested for culture	No of tissues with acid fast bacilli	No of animal tissues positive for NTM	No of animal tissues positive for MTBC
Cattle (*Bos indicus*)	499	38 (7.6)	36 (7.2)	2 (0.4)
African buffalo (*Syncerus caffer*)	55	5 (9.1)	3 (5.5)	2 (3.6)
African elephant (*Loxodonta africana*)	1	0 (0)	0 (0)	0 (0)
African civet (*Civettictis civetta*)	2	1 (0)	0 (0)	1 (50)
Lion (*Panthera leo*)	18	0 (0)	0 (0)	0 (0)
Black‐backed jackal (*Canis mesomelas*)^a^	1	0 (0)	0 (0)	0 (0)
Sported hyena (*Crocuta crocuta*)	1	0 (0)	0 (0)	0 (0)
Zebra (*Equus burchelli*)	1	0 (0)	0 (0)	0 (0)
Impala (*Aepxceros melampus*)^a^	4	0 (0)	0 (0)	0 (0)
Hartebeest (*Alceiaphus buselaphus*)		0 (0)	0 (0)	0 (0)
Baboon (*Papio anubis*)	1	1 (100)	1 (100)	0 (0)
Thompson gazelle (*Eudorcas thomsonii*)	7	2 (28.6)	2 (28.6)	0 (0)
Warthog (*Phacochoerus africanus*)	7	1 (14.3)	1 (14.3)	0 (0)
Honey badger (*Mellivora capensis*)	1	0 (0)	0 (0)	0 (0)
Klipspringer (*Oreotragus oreotragus*)	1	0 (0)	0 (0)	0 (0)
Waterbuck (*Kobus ellipsiprymnus*)	1	0 (0)	0 (0)	0 (0)
Wildebeest (*Connochaetes taurinus*)	1	0 (0)	0 (0)	0 (0)
Leopard (*Panthera pardus*)	3	0 (0)	0 (0)	0 (0)
Total	606	48 (7.9)	43 (7.09)	5 (0.83)

NTM, Non‐tuberculous mycobacteria, MTBC, Mycobacteria tuberculosis complex.

a Road kills = (Two (2) impala and one black‐backed jackal).

##### Processing animal tissues

Tissue samples of approximately 10 g were chopped into small pieces using sterile scalpel blades and forceps and transferred into stomacher bags containing 10 ml of sterile distilled water. The mixture was homogenized for 2 min using a blender stomacher machine (Stomacher 80 laboratory blender; Seward Medical, London, UK). The homogenates were transferred into universal containers, followed by addition of an equal amount of 3% oxalic acid. The suspensions were incubated for 45 min (Mwakapuja et al., 2013b) at room temperature with occasional shaking before being centrifuged at 3000 rpm (Mistral 1000 MSE; Fisher Scientific, Loughborough, UK) for 15 min. The sediments were neutralized with 3–4 drops of 2% sodium hydroxide solution to which 3–4 drops of 0.1% phenol red indicator were added to control the pH. The sediments were mixed before being inoculated on Lowenstein‐Jensen (LJ) media (BDH Chemicals Ltd, Poole, UK) containing pyruvate or glycerol and incubated at 37°C. Slopes were examined weekly for macroscopic growth of mycobacteria for the duration of 12 weeks. When growth was visible, smears were prepared, air‐dried, heat‐fixed, stained with Ziehl Neelsen (ZN) and examined microscopically for the presence of acid fast bacilli (AFB).

##### Sputum sample collection and preparation for culture

With assistance from hospital laboratory technicians, sputum samples were collected in 50‐ml screw‐cap Falcon tubes in the early morning from 472 patients who attended TB clinics in Serengeti (Mugumu), Bunda and Ngorongoro (Wasso) district‐designated hospitals (DDHs) and the Endulen Health Centre in NCA. All patients with symptoms suggestive of TB in the study area, who had not initiated anti‐tuberculosis treatment, were considered for inclusion in the study. Sputum digestion and decontamination was carried out using the method described by Pardini et al. (2005) where an equal volume of Cetyl Pyridinium Chloride (CPC) was added to the sputum samples. Thereafter, the samples were subsequently packed and transported by courier to the Muhimbili University of Health and Allied Sciences (MUHAS) TB laboratory in Dar es Salaam, Tanzania. The mixtures were left for 15 min at room temperature and concentrated by centrifuging at 4000 rpm for 15 min. The supernatants were poured off into a splash proof container. Twenty millilitres (20 ml) of sterile distilled water was added to the sediments, and the pellets were suspended by inverting the tubes several times before being centrifuged at 3500 rpm for 15 min. The supernatant was poured off, and then, the deposits were inoculated onto two slopes of LJ media containing either glycerol or pyruvate. The inoculated media were incubated at 37°C and examined weekly for bacterial growth up to 12 weeks. Slides of smears from the sediment were prepared for microscopy and thereafter stained by ZN method (Farnia et al., 2002).

### Extraction of DNA from sputum and tissue cultures

Colonies of each isolate were scraped from the surface of LJ medium (BDH Chemicals Ltd) and suspended into labelled cryovials containing 100 μl of sterile distilled water. The suspensions were heated in a water bath at 100°C for 1 h to inactivate the bacteria and then centrifuged at 3000 rpm (Mistral 1000 MSE) for 5 min. The supernatants were stored at −80°C until further analysis.

### Molecular identification of the genus *Mycobacterium* from human and animal isolates

Identification of the mycobacterium genus in animals was performed at SUA as described by Wilton and Cousins (1992) using six oligonucleotide primers. The 16S rRNA gene which is specific for mycobacterium genus was amplified using specific primers MYCGEN‐F (Fw) (5′‐AGA GTT TGA TCC TGG CTC AG‐3′) and MYCGEN‐R (Rev) (5′‐TGC ACA CAG GCC ACA AGG GA‐3′). Species from the *M. tuberculosis* complex were identified due to the two primers TB‐Fw (5′‐GAA CAA TCC GGA GTT GAC AA‐3′) and TB‐Rev (5′‐AGC ACG CTG TCA ATC ATG TA‐3′) that target the MPB70 gene specific for mycobacteria from the complex. Primers specific for a hypervariable region of the 16S rRNA gene of *Mycobacterium intracellulare* MYCINT‐Fw (5′‐CCT TTA GGC GCA TGT CTT TA‐3′) and *Mycobacterium avium* MYCAV‐Rev (5′‐ACC AGA AGA CAT GCG TCT TG‐3′) were included to amplify the two species. The PCRs were performed in total volume of 20 μl consisting of 10 μl HotStarTaq Master Mix (Qiagen, Manchester, UK), 6.2 μl nuclease‐free water (Qiagen), 0.3 μl of each oligonucleotide primer (100 μm) and 2 μl heat killed DNA template. PCR conditions for the reactions were as follows: denaturation was carried out at 95°C for 10 min followed by annealing, which involved 35 cycles for 1 min, 0.5 min and 1 min at 95, 61 and 72°C, respectively. Extension was done at 72°C for 10 min and cooling at 4°C infinitely at 4°C. PCR products were electrophoretically fractionated at 100 V in 1.5% agarose (Sigma^®^, St. Louis, MO, USA) gel and visualized after staining with ethidium bromide under UV light. The PCR products co‐electrophoresed with a hundred base pair (100 bp) DNA ladder, (Promega Corporation, St. Louis, MO, USA) to enable the calculation of the PCR product sizes. H_2_O was included as negative control and *M. tuberculosis* complex DNA as positive control.

### Identification of *Mycobacterium tuberculosis* complex

Deletion analysis by Gordon et al. (1999) and Mwakapuja et al. (2013b) was used to differentiate members of the *M. tuberculosis* complex as previously described. Two sets of primers, namely RD4 (RD4 Flank‐Fw (5ʹ‐CTC GTC GAA GGC CAC TAA AG‐3ʹ) and RD4 Flank‐Rev (5ʹ‐AAG GCG AAC AGA TTC AGCAT‐3′) and RD9 [RD9‐FlankFw (5ʹ‐AAC ACG GTC ACG TTG TCG TG‐3ʹ) and RD9‐InternalRev (5ʹ‐TTG CTT CCC CGG TTC GTC TG‐3ʹ)] were used during PCR amplification.

### Genotyping

Genotyping of mycobacterial DNA was carried out at the Centre of Excellence for Biomedical Tuberculosis Research/South African Medical Research Council (SAMRC) Centre for Molecular and Cellular Biology, Division of Molecular Biology and Human Genetics, Stellenbosch University in South Africa. Spoligotyping was performed by hybridization to 43 spacer oligonucleotides immobilized on a membrane as previously described by Kamerbeek et al. (1997). For MIRU‐VNTR, standardized typing was performed based on 24 loci primers as described by Supply et al. (2006).

### Data analysis

#### Spoligotype analysis

*M. bovis* spoligotypes were entered and validated in an Excel spreadsheet and then copied into authoritative names for spoligotype patterns available at www.miru-vntrplus.org. where the spoligotype pattern code was identified. The similarity coefficients (S_AB_) of spoligotypes were calculated by the formula S_AB_= [number of bands shared between A and B]/[(number of bands in A) + (number of bands in B) – (number of bands shared between A and B)] as described by Schmid et al. (1990) and modified by Godfrey‐Faussett and Stoker (1992).

For the MIRU‐VNTR analysis, the number of tandem repeat units was determined by estimating the size of the amplified DNA fragments, in relation to the known size of the repeat unit within the targeted VNTR locus. Results expressed in digital format were used for phylogenetic analysis using a dendogram. This data allowed comparison of large numbers of strains for establishment of strain family closeness and identification of transmission profiles in the ecosystem. Each digit signifies the number of copies at a particular locus. Phylogenetic analysis and creation of dendograms were performed using miru‐vntrplus (www.miru-vntrplus.org) and seaview version 4 software; parsimony analysis bootstrap with 100 replicates, 20 steps, and 43 sites as to generate a categorical‐based NJ‐Tree dendrogram to enable comparison of strain genotypes.

#### Ethical consideration

This study was independently reviewed and approved by the Research Ethical Committee of the Muhimbili University of Health and Allied Sciences (Ref.MU/PGS/PhD/R/Vol.1), The National Institute for Medical Research Ethical Committee (NIMR) (Ref. No. NIMR/HQ/R.8a/Vol. IX/1299) and Tanzania Wildlife Research Institute (TAWIRI) under the Ministry of Natural Resources and Tourism (MNRT), Tanzania (Ref. No. HA 403/563/01/74). Written informed consent was obtained from human participants who were free to withdraw from the study.

## Results

Of the 606 animal tissue specimens (107 from wild animals and 499 from indigenous cattle), 48 (7.9%) were AFB positive: 43 (7.09%) were non‐tuberculous mycobacteria (NTM) (Table 1) of which 20 isolates were *M. intracellulare*. In this study, the contamination rate of mycobacterial growth was 3%. Of the 43 NTM species that were detected from animals (indigenous cattle and wild animals), 25, 11 and seven species were sampled from Serengeti district, Ngorongoro district and from wildlife species in the ecosystem, respectively. No NTM species was identified from tissues collected from indigenous cattle in Bunda district. Moreover, of the 472 human sputum samples, only 12 specimens (2.9%) were NTM positive. No *M. avium* species were isolated from either human or animal specimens. Five [(5) (0.83%)] isolates had RD9 and RD4 loci deleted and were therefore confirmed as *M. bovis* strain (Table 1), of which two were isolated from indigenous cattle in Ngorongoro district and three from wild animal species with tuberculous lesions in Maswa Game Reserve and Serengeti National Park.

Of the 3 *M. bovis* isolates, two were retrieved from African civet and buffalo, from lung lesions and associated lymph nodes in Seronera (Central part of SNP) (Table 2). In addition, one of the *M. bovis* was isolated in lung lesion from buffalo in the eastern part of Maswa Game Reserve (Part of the Serengeti ecosystem). No prior information was available on health status of wild animals found with tuberculous lesions. All 3 *M. bovis* isolates from wild animals had identical spoligotype patterns (Table 2). These *M. bovis* isolates lacked spacers 3, 4, 5, 6, 7, 9, 16, 39, 40, 41, 42 and 43 and were previously classified as SB0133 according to the spoligotype database (Mbovis.org).

**Table 2:**
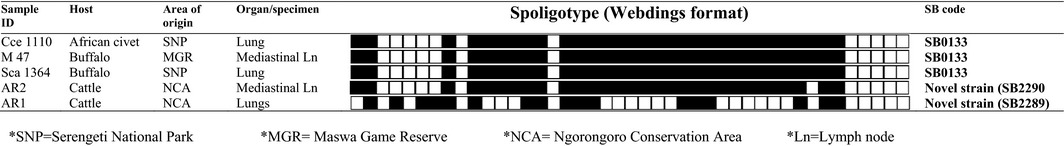
Spoligotype patterns of *M.  bovis* isolated from cattle and wildlife in the Serengeti ecosystem

All *M. bovis* isolates sampled from indigenous cattle (*Bos indicus*) were collected from Endulen and Olbalba villages in Ngorongoro Conservation Area, Ngorongoro district (Fig. 1). One *M. bovis* isolate (SB2290) from indigenous cattle showed a highly similar spoligotype to that seen in the wild animals with the exception that it lacked spacer 36. The spoligotype pattern from the remaining cow (SB2289‐sample ID; AR1) was characterized by absence of 26 spacers (Table 2). All of the *M. bovis* cultures had unique MIRU‐VNTR types (Fig. 3). Of the 472 culture isolates from sputum samples, 214 (45.3%) *M. tuberculosis* strains were isolated and no *M. bovis* was recovered. Other results regarding *M. tuberculosis* have been reported separately (Mbugi et al., 2015).

## Discussion

The aim of this study was to determine whether interspecies transmission of *M. bovis* at the human–livestock–wildlife interface of the Serengeti ecosystem exists by performing molecular characterization of *M.bovis* isolates. We found *M. bovis* at only a very low frequency in indigenous cattle and in wild animals but not in humans. Based on spoligotyping and phylogenetic analysis, all the *M. bovis* isolated from wild animals in Serengeti ecosystem were genetically related and belonged to spoligotype SB0133. Comparison of *M. bovis* isolated from wildlife with the two novel strains (SB2290 and SB2289) found in indigenous cattle had a spoligo patterns agreement of 96.8% and 45.2%, respectively. This indicates that at least some infections due to bovine tuberculosis (bTB) in the wildlife and indigenous cattle were genetically related.

**Figure 2:**
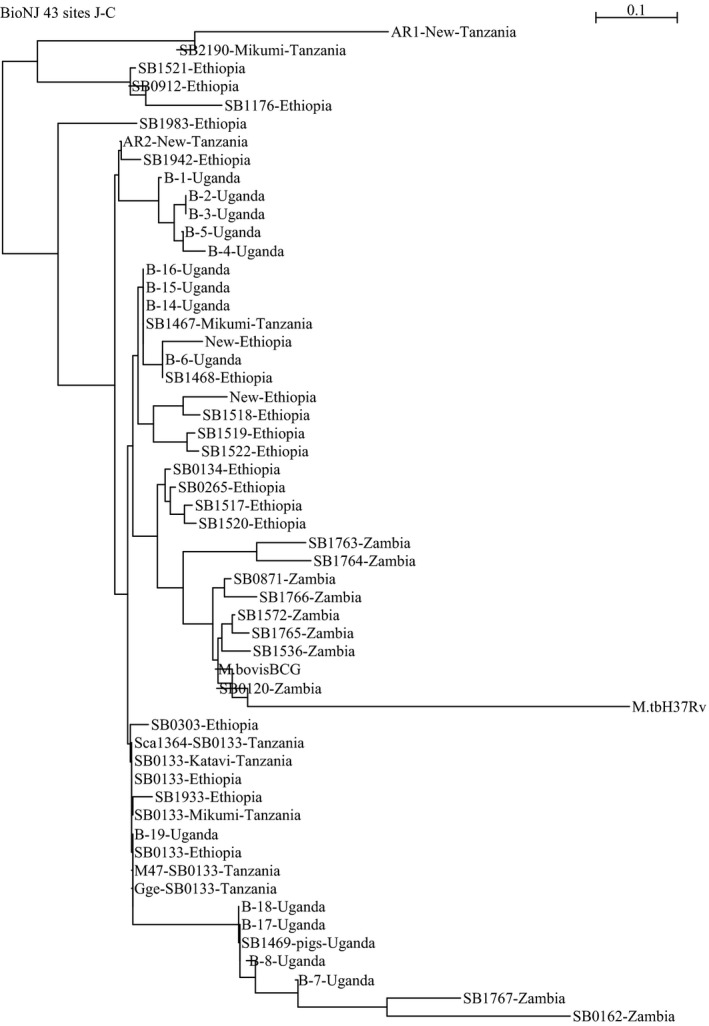
Phylogenetic tree based on spacer oligotyping of M. bovis strains sampled from Serengeti and other ecosystems in Tanzania (Clifford et al., 2013; Makondo, 2013; Mwakapuja et al., 2013b), Uganda (Oloya et al., 2007; Muwonge et al., 2012), Zambia (Munyeme et al., 2009) and Ethiopia (Biffa et al., 2010).

The spoligotype SB0133, which was dominant in wild animals (Cci1110, Sca1364 and M47), has also been isolated from cattle in the southern and northern parts of Tanzania (Berg et al., 2011; Mwakapuja et al., 2013b), indicating its widespread occurrence in the country. Moreover, the same strain has also been isolated from cattle in other countries including Ethiopia and Uganda (Oloya et al., 2007; Biffa et al., 2010), suggesting its wide distribution most possibly due to the movement of livestock and wild animals. Phylogenetic analysis of *M. bovis* indicated that a large diversity of strains is circulating in sub‐Saharan African countries. The novel strain (AR1) of *M. bovis* (SB2289) sampled from the Serengeti differs significantly from other strains in Tanzania, Uganda, Zambia and Ethiopia (Fig. 2). Other strains including the novel strain AR2 (SB2290) and SB0133 sampled in the Serengeti ecosystem relate genetically to *M. bovis* isolated from Tanzania, Uganda, Zambia and Ethiopia (Fig. 2). The spoligotype SB0133 isolated from wild animals in this study is the most common in East African region (Berg et al., 2011). The uniqueness of spoligotypes in this region may indicate evolutional change by the mycobacterium with adaptation of this spoligotype to the pastoral community (Biffa et al., 2010).

One of the novel *M. bovis* isolates from cattle (SB2290) differed to that found in wild animals by the loss of a single spacer (spacer number 36). This finding suggests that bTB infections in wild animals and cattle were epidemiologically related. Genetic diversity of *M. bovis* isolated from cattle in Ngorongoro might be attributed to extensive movement of cattle by herdsman searching for pasture and water during the dry season and intermingling of livestock in auction markets. It is believed that biological, ecological and anthropological processes are drivers for transmission of *M. bovis* at wildlife–livestock–human interfaces in sub‐Saharan Africa (de Garine‐Wichatitsky et al., 2013). Renwick et al. (2007) proposed that the rate of interspecies transmission which could occur at locations where domestic and wild animals congregate to rest, drink or feed is dependent on the interaction rate between the host species. This phenomenon is also encountered in other countries. In New Zealand, deer probably became infected through occasional direct contact with *M. bovis*‐infected cattle, or indirectly via shared feeding areas or pasture contamination (Nugent et al., 2015). In Spain, cattle and wild ungulates share the pastures and waterholes and both direct and indirect contacts must be frequent (Gortázar et al., 2008). However, further studies are required to establish contact rates and factors favouring *M. bovis* infection between cattle and wildlife and how this transmission takes place, and to eventually identify means to limit this risk (Gortázar et al., 2008).

**Figure 3:**
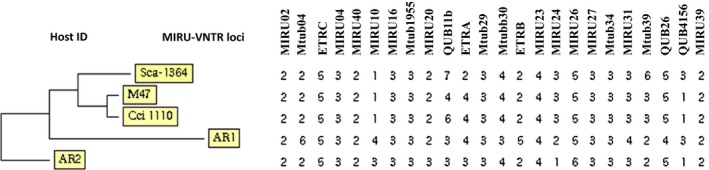
Phylogenetic relationship and 24 MIRU‐VNTR loci set variability of M. bovis isolated from cattle and wildlife.

Genetic relatedness of *M. bovis* isolates from African buffaloes and African civet (Table 2) sampled in the Maswa Game Reserve and Serengeti National Park, respectively, indicate the wide geographic spread of the SB0133 spoligotype (Fig. 2). However, spoligotype patterns of *M. bovis* from wild animals were distantly related to *M. bovis* from indigenous cattle (Fig. 3). Maswa Game Reserve and Serengeti National Park are part of the Serengeti ecosystem, with annual movements/migrations of ungulates particularly wildebeest and zebra driven by seasonal gradients of rainfall (Hopcraft, 2010). This is one of the risk factors that facilitates spread of pathogens particularly *M. bovis*, which can survive for several weeks in the environment (Aranaz et al., 2004). Isolation of *M. bovis* from wild animals in wildlife conservation areas does not only pose a threat to wild animals but also to the local economy that may be negatively impacted. Of particular concern is bTB infection in wildlife with far‐reaching consequences on, for example, biodiversity conservation, livestock production and tourism.

Further analysis of *M. bovis* isolates using MIRU‐VNTR revealed a high degree of polymorphism at the QUB 11b locus. Other MIRU‐VNTR loci such as ETR‐A, VNTR 3690, MIRU 24 and MIRU 10, MIRU2, MIRU4, MIRU16, MIRU23, MIRU27, MIRU39, MIRU40, VNTR 1955, ETR‐C, Mtub29 and Mtub34 showed lower resolution of MIRU‐VNTR typing. Collectively these findings signify that the QUB 11b locus has a higher discriminative power as compared to other VNTR loci and hence highlighting its importance in epidemiological investigation of *M. bovis*. Findings from this study are consistent with earlier studies conducted by Jeon et al. (2008), Skuce et al. (2002) and Smittipat et al. (2005) in which QUBs was more polymorphic than other MIRU‐VNTR loci. However, other studies conducted elsewhere have shown that QUB 11a, QUB 11b, QUB 18, ETR‐B and ETR‐C, Mtub 21, MIRU 16 and MIRU 26, ETR‐E, QUB 26, MIRU 23, ETR‐A and Mtub 12 were more polymorphic than other loci (Hlokwe et al., 2013). Previously, it has been shown that certain loci are useful in some countries but are considered of little value in others (Hlokwe et al., 2013).

Of particular note is the fact that all our *M. bovis* strains isolated were recovered from lungs and associated lymph nodes**,** suggesting aerosol or salivary transmission of bTB (Pritchard, 1988; Corner, 1994; Pollock et al., 2006; McNair et al., 2007). Besides finding *M. bovis*, we also isolated NTM in 7.09% of tissue samples collected from wild animals and livestock, which is consistent with our previous study conducted in the Serengeti ecosystem using Single Comparative Intradermal Tuberculin test (SCITT) that found the prevalence of non‐specific reactors to be 10.6% (Katale et al., 2013). Despite this high prevalence of NTM in the ecosystem, very little is known of their possible role in the formation of tuberculous lesions in which they were found. In their study, Katale et al. (2014) reported a diversity of NTM in the Serengeti ecosystem; some of them have pathogenic potential for humans and animals. *M. intracellulare*, part of the *Mycobacterium avium* complex (MAC), isolated from humans, livestock and wildlife was the most frequently isolated species followed by *M. lentiflavum* and *M. fortuitum*. *M. intra‐cellulare* has been associated with extrapulmonary and pulmonary TB in HIV individuals (Nyamogoba et al., 2012). Non‐tuberculous mycobacteria are opportunistic bacteria and have been associated with various clinical manifestations in human and animals. The isolation of NTM from tuberculosis‐like lesions in the absence of *Mycobacterium tuberculosis* complex (MTBC) calls for further research to elucidate their actual role in causing disease (Katale et al., 2014).

In this study, we did not find *M. bovis* in humans possibly due to the fact that we did not encounter cases of TB lymphadenitis as has been reported in other studies in Tanzania (Mfinanga et al., 2004), nor did we find co‐infection of *M. bovis* with NTM in culture isolates from animals. However, growth of *M. bovis* in LJ medium may have been concealed by the non‐tuberculous mycobacteria leading to a lower isolation frequency (Muwonge et al., 2012). This low isolation frequency is not uncommon as it has also been previously reported (Muwonge et al., 2012; Mwakapuja et al., 2013b). Moreover, the absence of zoonotic TB in humans could be due to the apparent low prevalence observed in indigenous cattle in the Serengeti ecosystem. In their study, Katale et al. (2013) reported 2.4% individual animal prevalence based on tuberculin testing. Despite the low isolation frequency of *M. bovis* strains from animal tissues, NTM was highly prevalent in this study as previously reported in other ecosystems in Tanzania, Uganda, Kenya and Ethiopia (Tschopp et al., 2011; Muwonge et al., 2012; Clifford et al., 2013; Gcebe et al., 2013; Makondo, 2013; Mwakapuja et al., 2013a; Mijele et al., 2014).

## Conclusion

This study found no clear evidence for recent cross‐species transmission of *M. bovis* between humans, livestock and wildlife at their interface. However, detection of *M. bovis* novel strains in indigenous cattle (*Bos indicus*) provides valuable epidemiological information concerning the transmission dynamics of *M. bovis* in wild animals and cattle population in the Serengeti ecosystem. In this study, very few *M. bovis* isolates were recovered from indigenous cattle and wild animal tissues. The few *M. bovis* isolates reported in this study could call for future longitudinal cross‐sectional studies to provide more information on the dynamics of *M. bovis* infection in this ecosystem.

Genetic relatedness of *M. bovis* in wild animals and indigenous cattle might suggest the existence of either evolutionary spillback of *M. bovis* infection from wild animal reservoirs to livestock or micro‐evolutionary events of *M. bovis* in cattle populations in the ecosystem. Another (not unlikely) possibility is that the observed genetic structure of *M. bovis* results from evolutionary events that take place in cattle populations outside the study area, possibly where the prevalence of the disease is higher, and that the *M. bovis* strains are merely imported into cattle and wild animal populations within the Serengeti system (Anonymous., 2015). Nevertheless, the very limited data presented in this study certainly do not clarify the transmission dynamics of *M. bovis* in the Serengeti ecosystem. We acknowledge the existing debate regarding the role of wild animals in bTB maintenance globally and in Africa, as current evidence is still not conclusive. Continued monitoring of *M. bovis* in both wild animals and livestock that may also include studies on the potential contribution of NTM in formation of tuberculous lesions is necessary.

## Conflict of Interest

The authors declare no conflict of interest.
